# Impact of myocardial deformation on risk prediction in patients following acute myocardial infarction

**DOI:** 10.3389/fcvm.2023.1199936

**Published:** 2023-08-10

**Authors:** Torben Lange, Roman J. Gertz, Alexander Schulz, Sören J. Backhaus, Ruben Evertz, Johannes T. Kowallick, Gerd Hasenfuß, Steffen Desch, Holger Thiele, Thomas Stiermaier, Ingo Eitel, Andreas Schuster

**Affiliations:** ^1^ Department of Cardiology and Pneumology, University Medical Center Göttingen, Georg-August University, Göttingen, Germany; ^2^ German Center for Cardiovascular Research (DZHK), Partner site Göttingen, Göttingen, Germany; ^3^ Institute for Diagnostic and Interventional Radiology, Faculty of Medicine and University Hospital Cologne, University of Cologne, Cologne, Germany; ^4^ School of Biomedical Engineering and Imaging Sciences, King’s College London, London, United Kingdom; ^5^ Institute for Diagnostic and Interventional Radiology, University Medical Center Göttingen, Georg-August University, Göttingen, Germany; ^6^ Department of Internal Medicine/Cardiology and Leipzig Heart Science, Heart Center Leipzig at University of Leipzig, Leipzig, Germany; ^7^ Medical Clinic II (Cardiology/Angiology/Intensive Care Medicine), University Heart Center Lübeck, University Hospital Schleswig-Holstein, Lübeck, Germany; ^8^ German Center for Cardiovascular Research (DZHK), Partner site Hamburg/Kiel/Lübeck, Lübeck, Germany

**Keywords:** cardiovascular magnetic resonance, myocardial feature tracking, strain analyses, risk prediction, acute myocardial infarct (AMI)

## Abstract

**Background:**

Strain analyses derived from cardiovascular magnetic resonance-feature tracking (CMR-FT) provide incremental prognostic benefit in patients sufferring from acute myocardial infarction (AMI). This study aims to evaluate and revalidate previously reported prognostic implications of comprehensive strain analyses in a large independent cohort of patients with ST-elevation myocardial infarction (STEMI).

**Methods:**

Overall, 566 STEMI patients enrolled in the CONDITIONING-LIPSIA trial including pre- and/or postconditioning treatment in addition to conventional percutaneous coronary intervention underwent CMR imaging in median 3 days after primary percutaneous coronary intervention. CMR-based left atrial (LA) reservoir (Es), conduit (Ee), and boosterpump (Ea) strain analyses, as well as left ventricular (LV) global longitudinal strain (GLS), circumferential strain (GCS), and radial strain (GRS) analyses were carried out. Previously identified cutoff values were revalidated for risk stratification. Major adverse cardiac events (MACE) comprising death, reinfarction, and new congestive heart failure were assessed within 12 months after the occurrence of the index event.

**Results:**

Both atrial and ventricular strain values were significantly reduced in patients with MACE (*p* < 0.01 for all). Predetermined LA and LV strain cutoffs enabled accurate risk assessment. All LA and LV strain values were associated with MACE on univariable regression modeling (*p* < 0.001 for all), with LA Es emerging as an independent predictor of MACE on multivariable regression modeling (HR 0.92, *p* = 0.033). Furthermore, LA Es provided an incremental prognostic value above LVEF (a c-index increase from 0.7 to 0.74, *p* = 0.03).

**Conclusion:**

External validation of CMR-FT-derived LA and LV strain evaluations confirmed the prognostic value of cardiac deformation assessment in STEMI patients. In the present study, LA strain parameters especially enabled further risk stratification and prognostic assessment over and above clinically established risk parameters.

**Clinical Trial Registration:**

ClinicalTrials.gov, identifier NCT02158468.

## Introduction

Patients suffering from acute myocardial infarction (AMI) are at high risk for recurrent major adverse cardiovascular events (MACE) despite optimized treatment and patient management (1). Many efforts to identify new parameters with greater prognostic benefits for improved risk stratification have been made over the past several years. In this context, cardiovascular magnetic resonance (CMR) imaging has evolved as a key imaging modality providing important information with incremental prognostic value (2). Particularly, CMR-feature tracking (CMR-FT)-derived left ventricular (LV) global longitudinal strain (GLS) and left atrial (LA) reservoir strain (Es) have been demonstrated to possess decisive diagnostic and prognostic capabilities, making them increasingly important for comprehensive cardiac performance analyses and optimized risk assessment (3–6). Importantly, besides their role as potent and superior prognostic parameters, various cutoff values for these strain measurements have been identified previously, enabling substantially improved risk stratification in patients suffering from AMI (5–7).

However, although the results of preceding studies are promising, a successful clinical application of these parameters and novel risk prediction models require independent validation (8). Only if previously identified imaging biomarkers and developed models can be utilized and proved to possess similar prognostic capabilities in external patient populations, a transfer of the findings to a widespread clinical practice or even guideline recommendations can be considered (9).

Whether previously determined cutoff values and associated prognostic benefits of atrial and ventricular strain values are applicable and can be reconfirmed in an independent cohort of STEMI patients is unknown. Therefore, the aim of this study is to reassess and revalidate the prognostic significance of CMR-FT-derived atrial and ventricular strain analyses in a large cohort of STEMI patients.

## Materials and methods

### Study population

The study population consisted of STEMI patients, who were enrolled in the LIPSIA CONDITIONING trial (identifier: NCT02158468), which was a prospective, randomized, open-label, controlled trial conducted at the University of Leipzig—Heart Center between April 2011 and May 2014. In this trial, STEMI patients undergoing primary percutaneous coronary intervention (PCI) were randomized in a 1:1:1 ratio to (1) combined intrahospital remote ischemic conditioning (RIC) and postconditioning (PostC) in addition to PCI; (2) PostC alone in addition to PCI; or (3) conventional PCI. Briefly, the combination of intrahospital RIC and PostC, in addition to primary PCI, significantly reduced the rate of MACE compared with conventional PCI, whereas sole PoctC, in addition to PCI, showed less distinctive and non-significant effects on MACE reduction. More information and a detailed study protocol have been published previously (10, 11). This study complied with the principles of Good Clinical Practice and the Declaration of Helsinki. The study protocol was approved by the local ethics committee and all patients gave written informed consent before participation.

### CMR imaging protocol

Between days 2 and 5 after the occurrence of the index event, all patients underwent an identical CMR imaging protocol on clinical 1.5 or 3.0 Tesla scanners for the assessment of infarct size (IS), presence and extent of microvascular obstruction (MO), myocardial salvage, volumetric analyses, and CMR-FT measurements. Balanced steady-state free precession (bSSFP) images were acquired in long axis (LAX) 2- and 4-chamber views (CV) as well as in short axis (SAX) orientation. The typical SSFP sequence parameters were as follows: repetition time 3.2 ms, echo time 1.2 ms, flip angle 60°, and 8 mm slice thickness in SAX. The exclusion criteria for CMR imaging comprised typical contraindications for CMR as described previously (12). More details on the CMR scan protocol have been published previously (13, 14). Intra- and interobserver analyses were performed including 30 randomly selected patients.

### CMR image analysis

**Figure 1:**
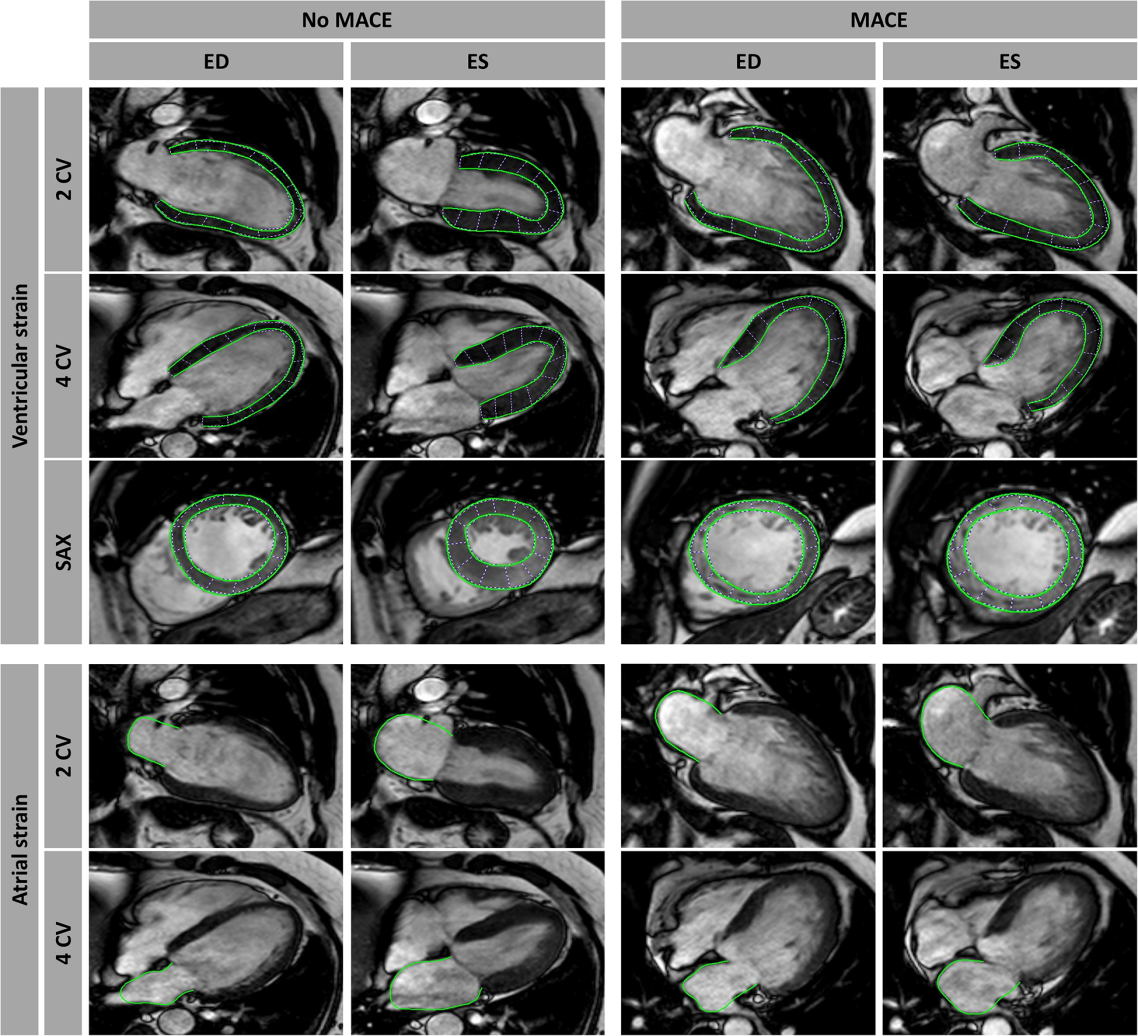
Cardiovascular magnetic resonance-feature tracking (CMR-FT). CMR-FT analyses in a patient with and without a major adverse cardiac event (MACE). Ventricular analyses were performed in long-axis 2- and 4-chamber views (CV) as well as in short-axis (SAX) stacks including basal, mid-ventricular, and apical slices, with only an exemplary mid-ventricular slice illustrated in this figure. Atrial strain analyses were performed in long-axis 2- and 4 CV images as well. Myocardial border delineations are presented in end diastole (ED) and end systole (ES).

Characteristics of myocardial infarction including IS, MO, and myocardial salvage, were assessed as previously described (14). CMR-FT was performed in a reputed imaging core laboratory with proven excellent reproducibility at the University Medical Center Göttingen (5, 15). Dedicated postprocessing software that has been validated and previously used in numerous studies (2D CPA MR, Cardiac Performance Analysis, Version 1.1.2, TomTec Imaging Systems, Unterschleissheim, Germany) (15–18) was used. For LV global longitudinal strain (GLS) analyses, myocardial borders were delineated in 2- and 4-CV images, whereas LV global circumferential strain and radial strain (GCS and GRS) evaluations were performed on the basal, midventricular, and apical levels of SAX images (Figure 1). Likewise, atrial strain assessments were conducted in 2- and 4-CV images comprising three atrial functional components: (1) reservoir function (Es) representing the collection of pulmonary venous return during ventricular systole, (2) conduit function (Ee) during passive passage of blood to the left ventricle during early diastole, and (3) boosterpump function (Ea) as the augmentation of ventricular filling during late ventricular diastole by active atrial contraction (19). After manual myocardial border delineation, the software's automated tracking algorithm was applied and accurate tracking was assured by a visual review of the contours with a reapplication of the algorithm after additional manual adjustments if necessary. All strain measurements were based on the average of three repeated and independent analyses.

### Clinical endpoints and outcome

The primary clinical endpoint of this study was a composite of all-cause death, reinfarction, and new congestive heart failure within 12 months after the occurrence of the index event. Blinded investigators collected the data and recorded these in standardized case report forms. A clinical events committee blinded to the assigned treatment adjudicated all components of documented clinical endpoints*.* Each patient contributed only once to the composite endpoint to avoid double counting. In case of the occurrence of multiple MACE, a priorization was made as follows: death > reinfarction > congestive heart failure. More detailed definitions on outcome assessments have been presented previously (11).

### Statistics

Baseline characteristics and CMR findings were reported according to the presence or absence of MACE. Categorical variables were presented as frequencies and percentages. Continuous variables were non-normally distributed as defined by the Shapiro–Wilk test and were provided as median with interquartile range (IQR). Intra- and interobserver variability was calculated using intraclass correlation coefficients (ICC) with a model of absolute agreement as well as the coefficient of variation (CoV), defined as the standard deviation of the differences divided by the mean. Correlations between LVEF and IS with CMR-derived strain values were analyzed using the Spearman method. Comparisons between groups were assessed by using the chi-square test for categorical variables and by using the nonparametric Mann–Whitney *U* test for continuous data. The Kaplan–Meier method was applied to analyze the occurrence of MACE between predefined subgroups, and differences were assessed by using the log-rank test. For LVEF, subgroups with ≤35% and >35% were classified according to current guideline recommendations. Furthermore, previously determined cutoff values for LV GLS (−16.4%), GCS (−21.0%), and GRS (20.3%) as well as LA Es (18.8%), Ee (10.1%), and Ea (10.3%) were used to further stratify patients into different risk groups (5, 6). Univariable and stepwise multivariate hazard ratios (HRs) with 95% confidence intervals (CIs) were calculated on the basis of Cox regression analyses to identify the predictors of MACE. To avoid statistical overfitting, multivariable models including a maximum of four parameters were developed. For assessing the predictive value of different strain measurements, the area under the curve (AUC) of the receiver operating characteristic curves was calculated. The results of C-statistics were compared using the non-parametric method devised by De Long et al., which has been previously described (20). The provided *p*-values are two-sided with an alpha level <0.05 considered statistically significant. IBM SPSS Statistic Software Version 28 (International Business Machines, Armonk, New York, USA) was used for statistical analyses.

## Results

### Patient characteristics

**Figure 2:**
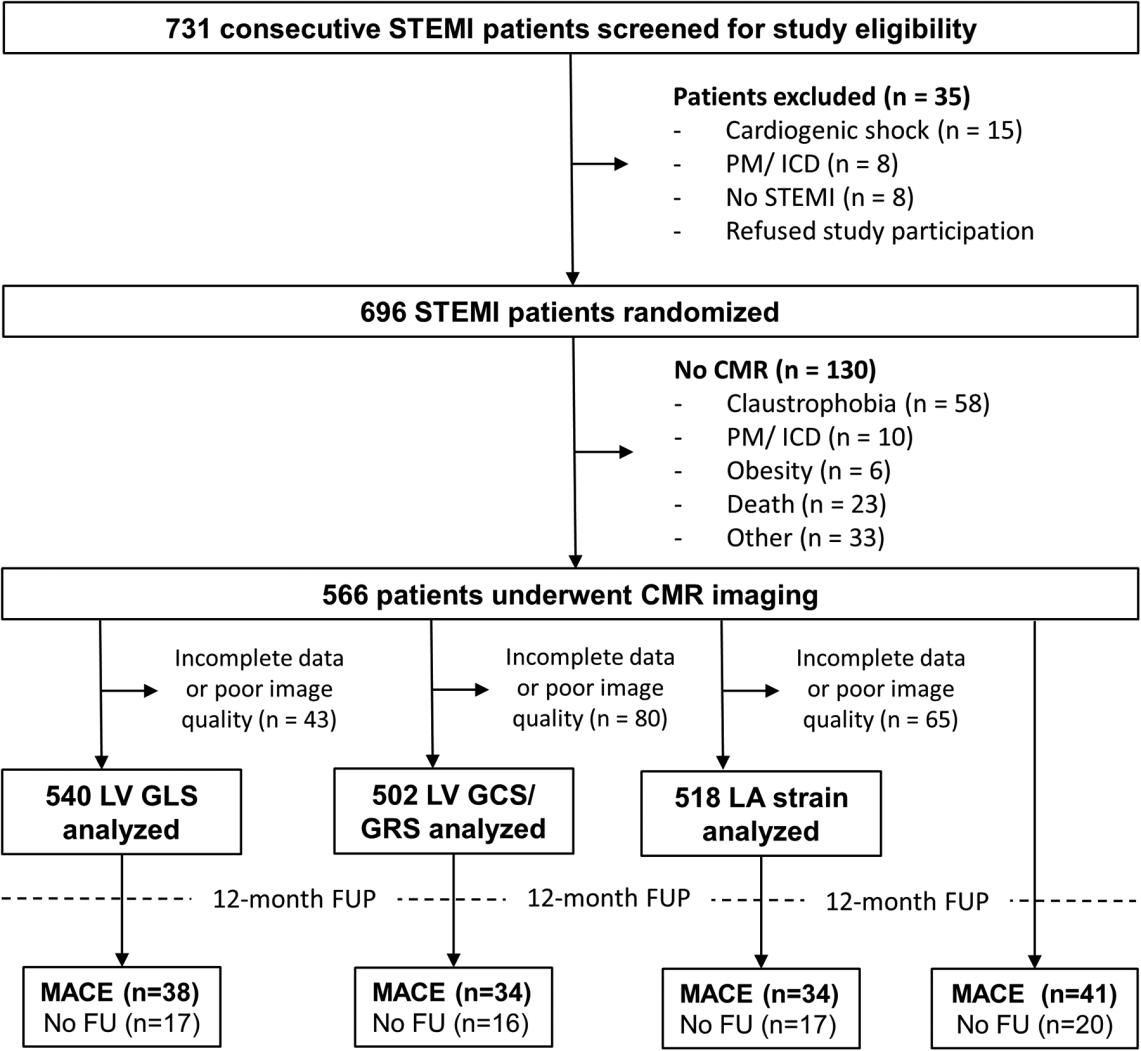
Study flowchart. A total of 696 of 731 eligible patients with ST-segment elevation myocardial infarction (STEMI) undergoing primary percutaneous coronary intervention (PCI) were enrolled within this study and 566 patients underwent cardiovascular magnetic resonance (CMR) imaging. ICD, implantable cardioverter defibrillator; GLS, global longitudinal strain; GCS, global circumferential strain; GRS, global radial strain; LA, left atrial; LV, left ventricular; MACE, major adverse cardiac events; PM, pacemaker.

Of the 696 enrolled STEMI patients, 566 patients underwent CMR imaging (Figure 2). A detailed overview of the baseline characteristics of the total cohort and their association with MACE is presented in Table 1. The median age of the study cohort was 63 years (54–74) with predominantly male patients (74.9%). Patients experiencing MACE were significantly older than those who did not experience MACE (70 years [58–77] vs. 57 years [46–71], *p* = 0.04). Patients with MACE had a significantly higher Killip class on admission (*p* = 0.001). There was no difference in cardiovascular risk factors between patients with and without MACE.

**Table 1:** Baseline characteristics.

Variables	All patients	MACE	No MACE	*p*-Value
	(*n* = 546)	(*n* = 41)	(*n* = 505)	
Age, years	63 (54–74)	70 (58–77)	57 (46–71)	**0.04**
Sex (male)	409 (74.9)	27 (65.9)	382 (75.6)	0.17
Cardiovascular risk factors				
Active smoking	235 (43.0)	15 (36.6)	220 (43.6)	0.39
Hypertension	402 (73.6)	32 (78.0)	370 (73.3)	0.5
Hyperlipoproteinemia	250 (45.8)	22 (53.6)	228 (45.1)	0.29
Diabetes	113 (20.7)	12 (29.3)	101 (20.0)	0.16
Body mass index (kg/m^2^)	27.1 (25.0–31.0)	28.2 (25.0–32.0)	27.0 (25.0–31.0)	0.66
Previous myocardial infarction	48 (8.8)	7 (17.1)	41 (8.1)	**0**.**013**
Previous coronary artery bypass graft	48 (8.8)	8 (19.5)	40 (7.9)	**0**.**001**
Anterior infarction	258 (47.3)	25 (61.0)	233 (46.1)	0.07
Killip class on admission				**0**.**001**
1	492 (90.1)	31 (75.6)	461 (91.3)	
2	38 (7.0)	6 (14.6)	32 (6.3)	
3	6 (1.1)	2 (4.9)	4 (0.8)	
4	10 (1.8)	2 (4.9)	8 (1.6)	
Door-to-balloon time (min)	26.0 (21.0–32.0)	26.0 (22.5–35.5)	26.0 (20.0–32.0)	0.26
Infarct-related artery				0.07
Left anterior descending	256 (46.9)	23 (56.1)	233 (46.1)	
Left circumflex	61 (11.2)	2 (4.9)	59 (11.7)	
Right coronary artery	229 (41.9)	16 (39.0)	213 (42.2)	
TIMI flow grade before PCI				0.44
0	306 (56)	21 (51.2)	285 (56.4)	
1	52 (9.5)	5 (12.2)	47 (9.3)	
2	117 (21.4)	7 (17.1)	110 (21.8)	
3	71 (13.0)	8 (19.5)	63 (12.5)	
TIMI flow grade after PCI				0.55
0	5 (1.0)	1 (2.4)	4 (0.8)	
1	6 (1.1)	0 (0)	6 (1.2)	
2	53 (9.7)	5 (12.2)	48 (9.5)	
3	482 (88.3)	35 (85.4)	447 (88.5)	
Medication				
Aspirin	545 (99.8)	40 (97.6)	505 (100)	
Clopidogrel/Prasugrel/Ticagrelor	546 (100)	41 (100)	505 (100)	1.0
Betablocker	535 (98.0)	40 (97.6)	495 (98.0)	0.84
ACE-inhibitor/AT-1 antagonist	533 (97.6)	40 (97.6)	493 (97.6)	0.98
Aldosterone antagonist	91 (16.7)	16 (39.0)	75 (14.9)	**<0.001**
Statin	540 (98.9)	39 (95.1)	491 (97.2)	0.44

Data are presented as *n* (in % of *N*) or median (interquartile range). For comparison of patients with and without MACE, *p*-values were calculated; the bold numbers indicate a statistically significant difference. The Mann–Whitney *U* test was used for testing continuous variables, and categorical variables were tested using the chi square test.

MACE, major adverse cardiac event; PCI, percutaneous coronary intervention, TIMI, thrombolysis in myocardial infarction.

### CMR results and outcome

At a 12-month follow-up, 41 MACE were documented (all-cause death = 19, reinfarction = 9, congestive heart failure = 13). CMR-derived infarct characteristics, as well as the results of CMR-FT strain measurements, are presented in Table 2. The results of intra- and interobserver analyses are provided in Supplementary Table S1. There were no statistical differences in strain values between the conditioning subgroups (Supplementary Table S2). Patients with MACE had a significantly larger IS (21.2% LV mass [13.5–37.3] vs. 17.1 [7.8–26.5], *p* = 0.035) and a smaller myocardial salvage and salvage index (7.8% LV mass [3.4–14.1] vs. 13.7 [7.2–19.7], *p* = 0.001 and 24.9 [11.3–39.2] vs. 45.1 [23.9–70.8], *p* < 0.001), whereas there was no difference in MO between patients with and without MACE (*p* = 0.12). Both ventricular and atrial strain values were significantly decreased in patients with MACE compared with those without (Table 2). All CMR-derived strain parameters correlated significantly with LVEF and IS (*p* < 0.001 for all), except LA Ea and IS (*p* = 0.07) (Table 3).

**Table 2:** Cardiac magnetic resonance results.

	All patients	MACE	No MACE	*p*-Value
LVEF, %	49.0 (40.7–57.0)	38.4 (28.5–49.9)	49.7 (41.7–57.0)	**<0.001**
LV EDV, ml	139.0 (117.7–167.6)	146.6 (118.9–191.6)	139 (117–167)	0.12
LV ESV, ml	70.0 (55.0–92.0)	95.0 (61–126.4)	69.0 (55–90.2)	**<0.001**
LV SV, ml	67.9 (54.9–81.8)	56.0 (44–79.6)	68.6 (55.8–82)	**0.01**
Infarct size, % LV mass	17.3 (8.2–26.8)	21.2 (13.5–37.3)	17.1 (7.8–26.5)	**0.035**
Microvascular obstruction, % LV mass	0 (0–1.8)	0.7 (0–3.4)	0 (0–1.7)	0.12
Myocardial salvage, % LV mass	13.4 (6.8–19.4)	7.8 (3.4–14.1)	13.7 (7.2–19.7)	**0.001**
Myocardial salvage index	43.6 (23.3–70.6)	24.9 (11.3–39.2)	45.1 (23.9–70.8)	**<0.001**
LV GLS, %	−16.3 (−12.4–−20.8)	−11.7 (−7.2–−20.1)	−16.5 (−12.8–−20.8)	**<0.001**
LV GCS, %	−20.8 (−16.7–−25.1)	−16.6 (−11.6–−21.7)	−21.0 (−17.2–−25.6)	**<0.001**
LV GRS, %	22.2 (16.9–27.7)	18.1 (11.1–21.6)	22.5 (17.2–27.8)	**<0.001**
LA reservoir strain, %	20.5 (16.2–25.1)	14.1 (9.8–19.4)	21.0 (16.6–25.1)	**<0.001**
LA conduit strain, %	8.6 (5.7–12.1)	5.2 (3.2–8.3)	8.7 (5.9–12.1)	**<0.001**
LA Boosterpump strain, %	11.7 (8.7–14.8)	9.4 (4.9–13.0)	11.8 (8.8–14.9)	**0.002**

Values are displayed as median (interquartile range). *P*-values were calculated for comparing patients with and without MACE using the Mann–Whitney *U* test. The numbers in bold indicate a statistically significant difference.

EDV, end-diastolic volume; ESV, end-systolic volume; GLS, global longitudinal strain; GCS, global circumferential strain; GRS, global radial strain; LA, left atrial; LVEF, left ventricular ejection fraction.

**Table 3:** Correlations of CMR-FT-derived strain values.

Variable	LVEF, %		Infarct size, % LV	
	Spearmans Rho	*p*-value	Spearmans Rho	*p*-Value
LV GLS, %	−0.58	**<0.001**	0.43	**<0.001**
LV GCS, %	−0.75	**<0.001**	0.51	**<0.001**
LV GRS, %	0.59	**<0.001**	−0.33	**<0.001**
LA reservoir strain, %	0.34	**<0.001**	−0.21	**<0.001**
LA conduit strain, %	0.37	**<0.001**	−0.25	**<0.001**
LA Boosterpump strain, %	0.17	**<0.001**	−0.09	0.07

The Spearman method was used for calculating correlations. The *P*-values in bold indicate a statistically significant correlation.

GLS, global longitudinal strain; GCS, global circumferential strain; GRS, global radial strain; LA, left atrial; LVEF, left ventricular ejection fraction.

In univariable Cox regression analyses, all LV and LA strain parameters were found to be associated with MACE (Table 4). Only significant predictors of MACE in univariable Cox regression analyses were included in multivariable calculations (Table 5). In multivariable models, LA Es emerged as the only independent strain value associated with MACE even after adjustment for risk factors, infarct characteristics, or LV strain measurements [HR 0.92 (95% CI 0.85–0.99), *p* = 0.033].

**Table 4:** Univariable Cox regression analyses.

Variables	Univariable	*p*-Value
	Hazard ratio (95% CI)	
Age	1.06 (1.03–1.08)	**<0.001**
Smoking	0.58 (0.35–0.94)	**0.028**
Diabetes	1.9 (1.2–3.1)	**0.007**
Kilip class on admission	1.9 (1.5–2.3)	**<0.001**
TIMI flow post PCI	0.6 (0.45–0.89	**<0.001**
Troponin	1.0 (1.0–1.0)	**0.046**
Infarct size	1.0 (1.0–1.1)	**0.003**
Microvascular obstruction	1.1 (1.0–1.1)	**0.015**
Myocardial salvage index	0.98 (0.96–1.0)	**<0.001**
LVEF	0.94 (0.91–0.96)	**<0.001**
LV EDV	1.0 (1.0–1.01)	**0.017**
LV ESV	1.0 (1.0–1.02)	**<0.001**
LV GLS	1.1 (1.1–1.2)	**<0.001**
LV GCS	1.1 (1.1–1.2)	**<0.001**
LV GRS	0.89 (0.84–0.94)	**<0.001**
LA Es	0.89 (0.84–0.94)	**<0.001**
LA Ee	0.89 (0.83–0.95)	**<0.001**
LA Ea	0.86 (0.81–0.95)	**<0.001**

Es, Reservoir strain; Ee, conduit strain; Ea, boosterpump strain; EDV, End-diastolic volume; ESV, end-systolic volume; GLS, global longitudinal strain; GCS, global circumferential strain; GRS, global radial strain; LA, left atrial; LVEF, left ventricular ejection fraction; PCI, percutaneous coronary intervention.

The *p*-values in bold indicate a statistically significant association with MACE.

**Table 5:** Multivariable Cox regression analyses.

Variables	Multivariable	*p*-Value
	Hazard ratio (95% CI)	
LA Es	0.92 (0.87–0.97)	**0** **.** **004**
LVEF	0.96 (0.93–0.99)	**0**.**01**
Troponin	1.0 (1.0–1.0)	0.74
LA Es	0.92 (0.87–0.97)	**0**.**004**
LV GLS	1.0 (0.93–0.99)	0.84
LVEF	0.96 (0.93–0.99)	**0**.**016**
LA Es	0.92 (0.87–0.98)	**0**.**008**
LV GCS	1.06 (0.96–1.18)	0.24
LVEF	0.98 (0.87–0.98)	0.46
LA Es	0.92 (0.87–0.98)	**0**.**006**
LV GLS	1.04 (0.96–1.1)	0.35
Infarct size	1.02 (0.96–1.05)	0.38
Microvascular obstruction	1.03 (0.96–1.1)	0.44
LA Es	0.92 (0.87–0.98)	**0**.**007**
LV GLS	1.0 (0.93–1.08)	0.98
LVEF	0.96 (0.93–0.99)	**0**.**023**
Kilip class on admission	1.5 (1.03–2.3)	**0**.**037**
LA Es	0.89 (0.85–0.94)	**<0.001**
Smoking	1.17 (0.58–2.4)	0.67
Diabetes	1.0 (0.45–2.23)	1.0
Kilip class on admission	1.6 (1.08–2.47)	**0**.**02**

Es, Reservoir strain; GLS, global longitudinal strain; GCS, global circumferential strain; LA, left atrial; LVEF, left ventricular ejection fraction.

The *p*-values in bold indicate a statistically significant association with MACE.

C-statistics revealed similar AUC values for LV GLS (AUC 0.68), LA Es (AUC 0.73), and LVEF (AUC 0.70). The addition of LA Es to LVEF resulted in a significant increase of C-statistics for MACE prediction compared with sole LVEF evaluation (Es + LVEF 0.74 vs. LVEF 0.70, *p* = 0.03).

**Figure 3:**
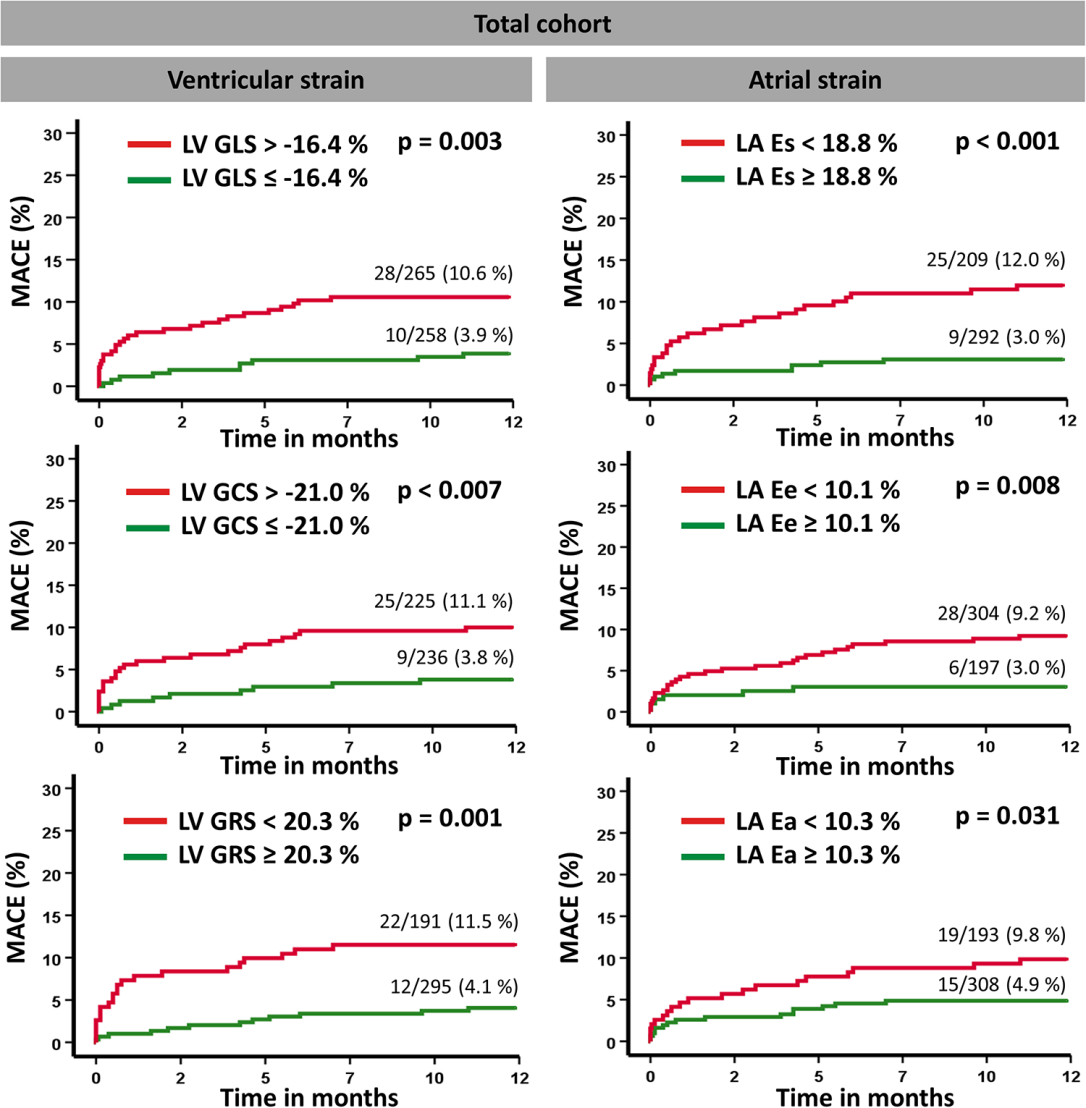
Kaplan–Meier survival curves. Event-free survival according to predefined cutoff values for left vertricular global longitudinal strain (GLS), circumferential strain (GCS), and radial strain (GRS) as well as left atrial reservoir (Es), conduit (Ee), and boosterpump (Ea) strain measurements with regard to the occurrence of a major adverse clinical event (MACE). A log-rank test was performed to compare the classified subgroups.

**Figure 4:**
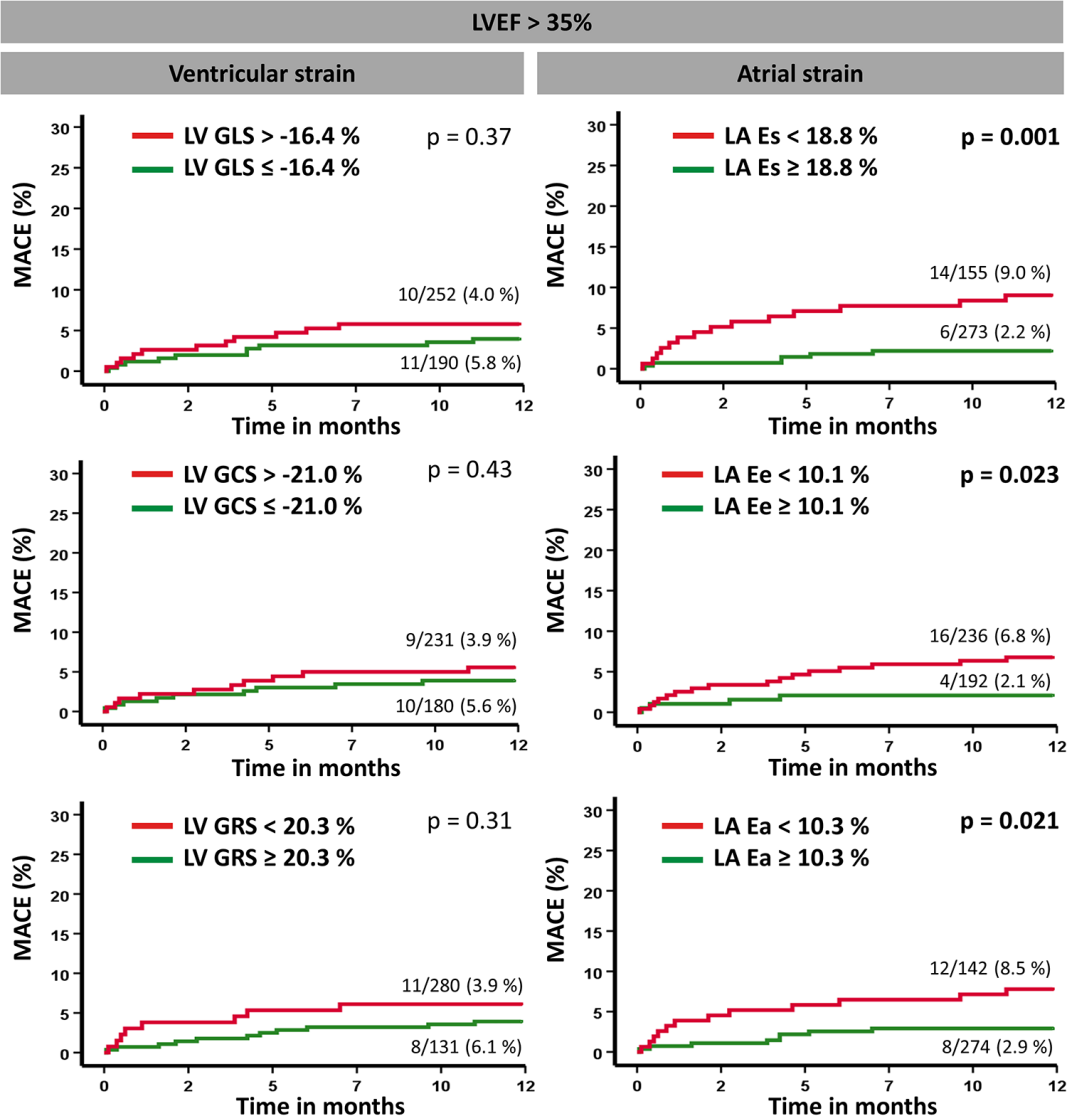
Kaplan–Meier survival curves according to LVEF >35%. Event-free survival according to predefined cutoff values for left ventricular global longitudinal strain (GLS), circumferential strain (GCS), and radial strain (GRS) as well as left atrial reservoir (Es), conduit (Ee), and boosterpump (Ea) strain measurements with regard to the occurrence of a major adverse clinical event (MACE). A log-rank test was performed to compare the classified subgroups.

Kaplan–Meier plots illustrating the applicability of predefined cutoff values for LA and LV strain measurements are presented in Figure 3. By using the predetermined cutoff values for each strain parameter, a dichotomization in both high- and low-risk groups was feasible. Furthermore, among patients considered at relatively low risk by the parameter of LVEF >35%, the cutoff values for LA Es, Ee, and Ea allowed additional risk stratification by the identification of subgroups with a higher risk for MACE (Figure 4). Further dichotomization into subgroups according to LV strain cutoff values did not provide additional risk stratification on log-rank testing (GLS: *p* = 0.37, GCS: *p* = 0.43, GRS: *p* = 0.31). In high-risk patients with LVEF ≤35%, no further risk stratification was possible by the application of predefined cutoff values, except for LA Ee (Supplementary Figure S1).

## Discussion

This CMR imaging study aimed to assess and validate the prognostic importance and predefined cutoff values of comprehensive CMR-FT-derived strain analyses in an independent large cohort of STEMI patients. Several notable findings should be considered: (1) LV GLS, GCS, and GRS as well as LA Es, Ee, and Ea were significantly reduced in patients experiencing MACE during a 12-month follow-up. (2) LA Es emerged as the strongest predictor for MACE, outperforming all LV strain values (3) Applying predefined cutoff values of LA and LV strain measurements enabled accurate risk stratification, with especially LA strain values identifying additional high-risk patients beyond commonly used parameters.

Myocardial performance analyses are essential for optimal risk stratification and patient management after AMI (21). Based on a standard CMR imaging protocol not requiring additional scanning time, CMR-FT-derived strain values represent robust imaging biomarkers. Beyond myocardial volumetric evaluations, these strain measurements allow making conclusions not only on global cardiac function but also on regional levels (22) and can additionally provide insights into diastolic capacities (23). Importantly, comprehensive myocardial strain measurements have been repeatedly proved to be sensitive parameters for myocardial dysfunction, enabling improved risk assessment outperforming most commonly used LVEF (5, 24). However, after the identification of prognostic powerful strain parameters and development of novel risk prediction models in preceding studies, external and independent validations of the findings are a prerequisite for implementing these imaging biomarkers and their cutoff values as useful tools in clinical practice and for potential further therapeutic decision-making.

In line with previous findings, our study demonstrated all strain parameters to be significantly reduced in patients with MACE compared to those without MACE. Further, both LA and LV strain values were predictors of MACE in univariable regression calculations. However, after adjusting for univariably significant parameters in different models such as cardiac risk factors, LVEF, or IS characteristics, our study could not reconfirm LV strain parameters to be independent predictors of MACE when simultaneously including LVEF in multivariable regression models. These results are in contrast to several previous works including the derivation cohort for the cut-off values, that have shown a superior role for LV strain in patients following AMI (4, 5). Of note, these studies included larger AMI cohorts with >1,000 patients in their analyses. In particular, LV GLS has been demonstrated to possess an eminent and independent prognostic role not only above commonly used parameters such as LVEF but also among all strain measurements in a spectrum of various different cardiovascular diseases (3, 5, 7, 25–28). It remains unclear, if an absent superior prognostic role of LV strain values and especially LV GLS, might be caused by a smaller study population with a relatively too low event rate of 41 MACE compared with some of the previous studies, which might influence and hamper the validation and reconfirmation of earlier findings. It is noteworthy that in contrast to other AMI study cohorts at least parts a proportion of the current study population underwent RIC and PostC or only PostC in addition to conventional PCI. Although there were no significant differences between the conditioning subgroups and controls, one cannot exclude an additional influence on strain alterations after the coapplication of RIC or PostC. As a consequence, this could reduce the prognostic power of GLS values measured shortly after the occurrence of the acute index event. At present, there is a contrary debate in the literature pertaining to whether and to what extent the coapplication of RIC influences myocardial damage and outcome after AMI. While some studies have documented positive effects of RIC on myocardial salvage and outcome (10, 11, 29), others have documented neutral effects of RIC on CMR-derived IS or outcome (30). However, especially improved GLS values have been repeatedly shown after the coapplication of RIC (31, 32) and, therefore, similar positive effects of RIC resulting in substantial GLS improvement during follow-up might have influenced the results of our study. Consequently, these effects of an improved GLS during follow-up might at least partly distort the superior prognostic power of GLS in our study compared with the findings in other study cohorts. Moreover, one might speculate whether a lower prognostic power of GLS compared with previous findings might be biased by differing baseline characteristics or varying infarct properties and localizations (33). Nevertheless, relatively similar distribution patterns of the diseased vessels as well as infarct characteristics can be observed when comparing these parameters with those of preceding studies, which most likely does not explain the failed validation as an independent predictor of MACE in our work. Remarkably, there were no significant differences of cardiovascular risk factors between patients with an adverse event and those without MACE in our study cohort, which is in contrast to other study populations, and might be another component of explanation for a less powerful prognostic relevance of LV GLS in this study. However, previous studies evaluating large STEMI registries or pooled data from various AMI trials documented equal or even worse clinical outcomes in patients without cardiovascular risk factors compared with patients presenting with at least one cardiovascular risk factor (34, 35). Whether this risk factor distribution pattern may have influenced the predictive power of GLS analyses cannot be answered on the basis of our data. Lastly, whether a missing 3-CV long axis orientation within the CMR scan protocol might influence the prognostic value of GLS remains speculative.

In contrast, LA Es emerged as the only independently associated MACE predictor in a multivariable regression model including all LV strain parameters and, moreover, increased the diagnostic accuracy of MACE prediction in AUC analyses. These findings are in line with previous study findings (16, 36) and underline the important role of atrial deformation analyses in AMI patients. Although LA performance is inevitably associated with and influenced by LV function, LA deformation has the potential to compensate ventricular failure after AMI (37). Consequently, reduced LA mechanics (especially LA Es) are sensitive markers of atrial compliance and compensatory capacity with a significant association with cardiovascular risk. In this context, intrinsic atrial failure is increasingly discussed and considered as an atrial cardiomyopathy beyond being merely a consequence of LV failure (38). It is important to note, that LA Es also represent the cumulative adverse impact of impaired LA relaxation and pulmonary venous congestion, which allows risk prediction beyond LV systolic function assessment (39). Furthermore, it has been shown, that LA strain deterioration precedes ventricular strain alterations (40) and LA strain assessment even enables a more precise detection of diastolic dysfunction than invasive pressure measurements (41). Importantly, beyond a successful validation of predetermined LA and LV strain cutoff values for risk prediction in the present study cohort, LA strain cutoff values allowed a further identification of patients at higher risk, yielding substantial additional value for prognostic characterization beyond the established clinical markers of MACE.

Analyzing strain performance to identify high-risk patients following AMI entails several important clinical considerations. Beyond a more precise detection of mechanical dysfunction compared with the commonly used LVEF, the prognostic implications could enable important improvements in patient management. It is noteworthy that a large number of patients suffering sudden cardiac death after AMI were shown to have an LVEF above 35% (42). Therefore, since current guideline recommendations for implantable cardioverter-defibrillator indications almost entirely rely on LVEF assessment, future clinical decision-making might include impaired atrial strain measurements when considering patients for primary prevention device implantation despite a relatively preserved LVEF*.* Likewise, patients at higher risk according to LA strain deteriorations might face a more aggressive or tailored treatment for heart failure including pharmacotherapy and/or device therapy. It is noteworthy that with the approval of SGLT-2 inhibitors in the recent past with potential positive effects on diastolic and subsequently atrial function, the prognostic attributes, especially of atrial strain values, might change in the future.

Initial efforts including 3-dimensional analyses or using neuronal networks for automated and facilitated deformation assessment have been made with promising results (43, 44), and it is interesting to speculate whether future software refinements might further improve the diagnostic and prognostic accuracy of strain parameters in patients following AMI. Of note, vendor- or technique (e.g., myocardial tagging or SENC)-dependent differences in strain values need to be considered when interpreting strain parameters. Although similar reproducibility and prognostic values of strain parameters have been demonstrated using different postprocessing software or techniques (5, 24, 45, 46), the application of different software packages or techniques might hamper direct comparability of the parameters (47). In this context, further standardization of image acquisition and analysis is required to reduce systematic intervendor differences and to harmonize comparability (46, 48).

Despite these considerations, further analyses and larger validation studies in additional STEMI cohorts (undergoing additional RIC/PostC) with different cardiovascular risk factor distribution patterns and a larger number of events are required to establish these imaging biomarkers for widespread clinical use in AMI patients.

### Limitations

Compared with other studies that analyzed and validated strain values in AMI patients (4, 5), the current study cohort was considerably smaller. Furthermore, long-axis SSFP images were acquired only in 2- and 4-CV orientations but not in a 3-CV orientation. Patients with contraindications for CMR imaging (e.g., due to metallic implants or renal failure) and/or potentially sicker patients (e.g., those with cardiogenic shock or those unable to lie in a supine position during the conduct of the CMR scan protocol) were not included in the study, resulting in a selection bias of a potentially lower-risk population. In addition, an optimal time point for CMR imaging and deformation assessment after AMI is unknown. Consequently, CMR imaging and strain measurements taken after the occurrence of the index event could detect further strain alterations following reperfusion therapy, for example, those caused by myocardial remodeling processes and/or the effects of RIC/PostC. Therefore, additional CMR follow-up scans would be highly desirable to assess the temporal course of strain alterations after AMI and to potentially provide even better prognostic value of strain analyses.

## Conclusion

This study aimed to validate and reconfirm the incremental prognostic value of both CMR-FT-derived LV and LA strain parameters for risk stratification in an external population of patients following AMI. In line with the literature, all strain values were significantly reduced in patients with MACE. While LV strain assessment did not enable improved risk prediction compared with the commonly used LVEF, LA Es emerged as an independent and superior imaging marker providing important prognostic information beyond traditionally used clinical parameters. Larger validation studies are needed for an unlimited and widespread application of comprehensive strain analyses in clinical routine.

## Data Availability

The data that support the findings of this study are available from the corresponding author upon reasonable request.
