# Supplementary material for: Impact of myocardial deformation on risk prediction in patients following acute myocardial infarction
Source: Front Cardiovasc Med. 2023 Aug 10;10:1199936. doi: 10.3389/fcvm.2023.1199936 (PMC10449121; doi:10.3389/fcvm.2023.1199936)
Supplement: Supplementary file 1 [file Table1.docx]

**Supplementary Table 1: Intra- and inter-observer analyses**

|  | **Intra-observer** | | | **Inter-observer** | | |
| --- | --- | --- | --- | --- | --- | --- |
|  | Mean difference (SD of the diff.) | ICC (95% CI) | CoV (%) | Mean difference (SD of the diff.) | ICC (95% CI) | CoV (%) |
|  |  |  |  |  |  |  |
| **Left ventricle** |  |  |  |  |  |  |
| LV GLS [%] | -0.03 (0.82) | 0.995 (0.991–0.997) | 4.23 | 0.08 (0.69) | 0.997 (0.994–0.998) | 3.52 |
| LV GCS [%] | -0.24 (0.59) | 0.998 (0.996–0.999) | 2.43 | -0.01 (0.69) | 0.998 (0.996–0.999) | 2.83 |
| LV GRS [%] | -0.87 (2.79) | 0.943 (0.896–0.968) | 11.78 | -0.61 (2.69) | 0.943 (0.899–0.968) | 11.54 |
| **Left atrium** |  |  |  |  |  |  |
| Es [%] | 0.29 (1.01) | 0.993 (0.986–0.996) | 4.71 | 0.99 (2.14) | 0.962 (0.919–0.980) | 10.02 |
| Ee [%] | 0.34 (1.23) | 0.976 (0.956–0.986) | 11.51 | 0.27 (1.78) | 0.953 (0.918 –0.974) | 16.57 |
| Ea [%] | -0.05 (1.35) | 0.969 (0.949–0.982) | 12.52 | 0.72 (2.10) | 0.899 (0.815–0.944) | 19.81 |

CoV: coefficient of variation, Es: Reservoir strain, Ee: conduit strain, Ea: boosterpump strain, GLS: global longitudinal strain, GCS: global circumferential strain, GRS: global radial strain, ICC: intraclass correlation coefficient, SD: standard deviation

**Supplementary Table 2:** Cardiac magnetic resonance results according to treatment arms

| **Variable** | **RIC+PostC (n=180)** | **PostC only (n=198)** | **Control**  **(n=186)** | **RIC+PostC vs.**  **Control** | **PostC**  **vs.**  **Control** | **RIC+PostC**  **Vs.**  **PostC** |
| --- | --- | --- | --- | --- | --- | --- |
|  |  |  |  |  |  |  |
|  |  |  |  | ***p-value*** | | |
| **LVEF (%)** | 48.2 (40.5 – 56.6) | 49.8 (41.8 – 57.3) | 49.4 (39.2 – 56.6) | 0.72 | 0.4 | 0.21 |
| **LV GLS (%)** | -15.4 (-12.4 - -19.6) | -17.2 (-13.1 – 21.4) | -15.8 (-11.6 - -20.9) | 0.7 | 0.07 | **0.018** |
| **LV GCS (%)** | -20.5 (-16.3 - -24.7) | -22.1 (-17.2 - -25.6) | -20.1 (-16.7 - -24.9) | 0.51 | 0.14 | 0.38 |
| **LV GRS (%)** | 20.8 (16.1 – 27.1) | 23.2 (18.0 – 27.8) | 22.2 (16.6 – 26.0) | 0.6 | 0.17 | 0.08 |
| **LA Es (%)** | 19.4 (15.9 – 24.7) | 21.7 (16.4 – 24.8) | 20.2 (15.9 – 25.2) | 0.86 | 0.46 | 0.35 |
| **LA Ee (%)** | 8.2 (5.9 – 12.2) | 8.7 (5.9 – 12.2) | 8.8 (4.7 – 11.8) | 0.93 | 0.6 | 0.56 |
| **LA Ea (%)** | 11.1 (8.7 – 14.6) | 11.6 (9.5 – 14.7) | 11.9 (8.3 – 15.4) | 0.54 | 0.87 | 0.47 |

Values are displayed as median (interquartile range). P values were calculated for the comparison between subgroups using the Mann-Whitney U test. Numbers in bold indicate a statistical significance in difference. Es: Reservoir strain, Ee: conduit strain, Ea: boosterpump strain EDV: enddiastolic volume, ESV: endsystolic volume, GLS: global longitudinal strain, GCS: global circumferential strain, GRS: global radial strain, LA: left atrial, LVEF: left ventricular ejection fraction.

**
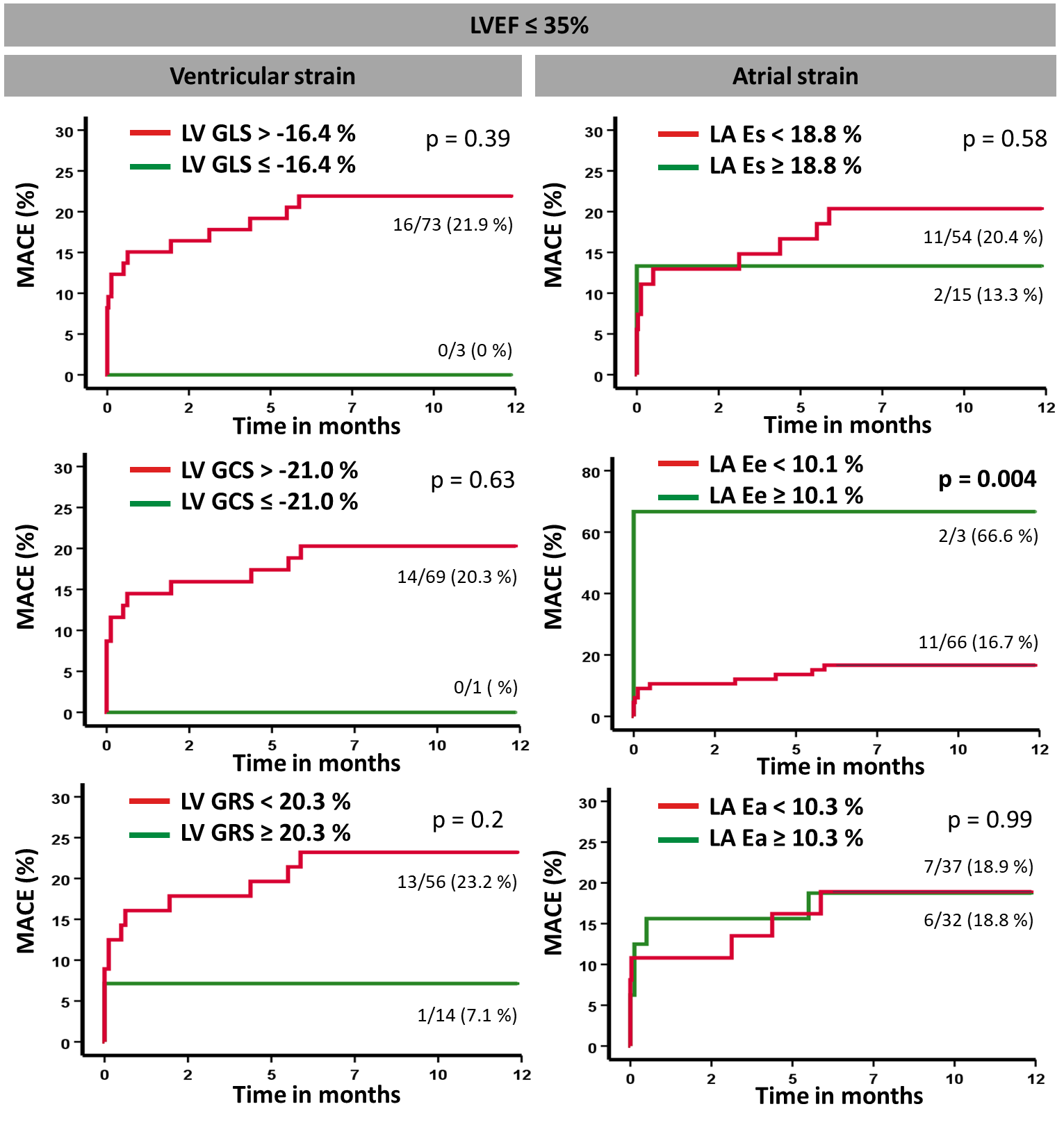
**

**Supplementary Figure 1: Kaplan Meier survival curves according to reduced LVEF ≤ 35%**

Event-free survival according to predefined cut-off values for left ventricular global longitudinal (GLS), circumferential (GCS) and radial strain (GRS) as well as left atrial reservoir (Es), conduit (Ee) and boosterpump (Ea) strain measurements regarding the occurrence of a major adverse clinical event (MACE). Log-rank testing was performed to compare classified subgroups.
